# Stem-Loop RNA Hairpins in Giant Viruses: Invading rRNA-Like Repeats and a Template Free RNA

**DOI:** 10.3389/fmicb.2018.00101

**Published:** 2018-02-01

**Authors:** Hervé Seligmann, Didier Raoult

**Affiliations:** Unité de Recherche sur les Maladies Infectieuses et Tropicales Emergentes, UMR MEPHI, Aix-Marseille Université, IRD, Assistance Publique-Hôpitaux de Marseille, Institut Hospitalo-Universitaire Méditerranée-Infection, Marseille, France

**Keywords:** systematic nucleotide exchange, swinger DNA polymerization, invertase, 3′-to-5′ polymerization, transcription, *Acanthamoeba castellanii*

## Abstract

We examine the hypothesis that *de novo* template-free RNAs still form spontaneously, as they did at the origins of life, invade modern genomes, contribute new genetic material. Previously, analyses of RNA secondary structures suggested that some RNAs resembling ancestral (t)RNAs formed recently *de novo*, other parasitic sequences cluster with rRNAs. Here positive control analyses of additional RNA secondary structures confirm ancestral and *de novo* statuses of RNA grouped according to secondary structure. Viroids with branched stems resemble *de novo* RNAs, rod-shaped viroids resemble rRNA secondary structures, independently of GC contents. 5′ UTR leading regions of West Nile and Dengue flavivirid viruses resemble *de novo* and rRNA structures, respectively. An RNA homologous with Megavirus, Dengue and West Nile genomes, copperhead snake microsatellites and levant cotton repeats, not templated by Mimivirus' genome, persists throughout Mimivirus' infection. Its secondary structure clusters with candidate *de novo* RNAs. The saltatory phyletic distribution and secondary structure of Mimivirus' peculiar RNA suggest occasional template-free polymerization of this sequence, rather than noncanonical transcriptions (swinger polymerization, posttranscriptional editing).

## Introduction

Diverse numbers of simple organic compounds spontaneously self-organized at life's origins. This system crystallized around the ribonucleic-protein system forming the main organizational building blocks of known organisms (Szostak, 2009; Ruiz-Mirazo et al., 2014). Then presumably the tRNA-rRNA information-storage/translation apparatus developed (Fox, 2010; Root-Bernstein and Root-Bernstein, 2015, 2016) through segment accretion (Di Giulio, 1992, 1994, 1995, 1999, 2008, 2009, 2012, 2013; Widmann et al., 2005; Branciamore and Di Giulio, 2011, 2012; Seligmann, 2014; Petrov et al., 2015). Presumably, self-replicating systems evolved, producing/parasitized by molecules lacking self-replication capacities (Bansho et al., 2012). The system potentially stabilized by evolving molecular cooperation between molecules with replicating capacities (Penny, 2015) and others lacking this capacity but contributing otherwise to the system's persistence (Higgs and Lehman, 2015). This process would have produced the modern translation/replication system(s).

Other molecules (short parasitic repeats, frequently forming stem-loop hairpins, viroids, viruses, etc.) presumably subsisted mainly as parasites and occasionally contributing new, sometimes functional parts. Persistence of the cooperative system implies integrating new molecules with new functions, while channeling most resources to critical components such as ribosomes. Nowadays, ribosomes compete with parasitic elements that hijack the cell's integrated cooperative system (Xie and Scully, 2017). Viruses frequently mimic cellular processes (Hiscox, 2007), including the cell's replication/transcription compartments (Chaikeeratisak et al., 2017). Hence when life began, ribosomal RNAs had virus-like properties. This arms race might explain why >95% of the cell's transcriptome consists of ribosomal RNA (Peano et al., 2013). Here we hypothesize that new RNAs still spontaneously emerge and integrate molecular cooperative systems of organisms.

We use several analyses, including comparisons among RNA secondary structures, to detect and test for *de novo* RNA emergence.

### Structural homology

**Figure 1:**
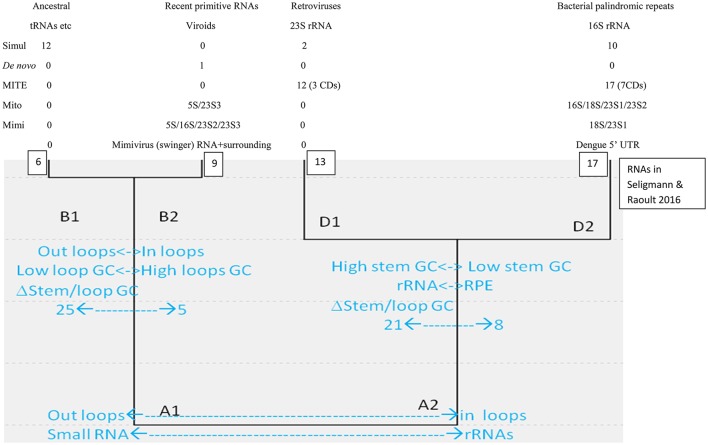
Hierarchical cluster of secondary structures formed by diverse RNA molecules, adapted from Seligmann and Raoult (2016).

Classical sequence homology between linear sequences is inefficient at reconstructing ancient evolution because sequences evolve relatively fast. Structures, rather than sequences, are conserved for longer periods. For example, analyses considering structural homology among proteins suggest a common cellular ancestry for modern cells and viruses (Nasir and Caetano-Anollés, 2015). Similarly, analyses using simple properties of secondary structures formed by diverse RNAs detect two main clusters (A1 and A2, small vs. complex RNAs), each subdivided into two main groups (A1:B1-B2; A2:D1-D2), schematized in Figure 1.

Cluster B1 is the functionally most diverse group and includes several presumably ancestral RNAs, such as replication origin, tRNA and some ribozymes. Its “sister” cluster B2 includes diverse, probably more derived/recent molecules (i.e., the only known protein-encoding viroid, AbouHaidar et al., 2014). Clusters D1 and D2 are characterized by rRNA subunits, associated with parasitic RNAs: D1 includes all six 23S rRNA subunits and retroviruses; and D2 groups all 16S rRNA subunits and most families of RPEs, rickettsial palindromic elements, which infest specifically *Rickettsia* genomes (Amiri et al., 2002; Gillespie et al., 2012). Secondary structure similarities between parasitic and ribosomal RNAs underscore virus-like rRNA properties (Figure 1), presumably due to the assumed arms race between rRNAs and parasitic RNAs.

Cluster D2 includes half of the secondary structures formed by tRNA sequences. Hence B1–D2 would represent organic life's main tRNA-rRNA axis of molecular evolution, where simple ancestral tRNA-like RNAs complexified into rRNA-like RNAs (Bloch et al., 1983, 1984, 1989). This interpretation is in line with detections of candidate tRNA genes within mitochondrial 16S rRNA of chaetognath mitogenomes that otherwise would lack tRNAs (Barthélémy and Seligmann, 2016).

Here we analyze additional types of RNAs and explore their similarities with clusters B1-2/D1-2. Additional viroids, are analyzed to explore the possibility that some viroids date from the precellular world (Bussière et al., 1995; Diener, 1996) and others emerged *de novo* recently (Koonin and Dolja, 2013; Seligmann and Raoult, 2016), potentially solving the conundrum about primordial/recent *de novo* viroid origins (Diener, 2016). Two RNA types function as controls to confirm the statuses of putative ancestral/*de novo* clusters B1 and B2. The remaining RNAs originate from giant viruses and test putative evolutionary links between giant viruses and rRNAs.

## Materials and methods

### Secondary structure predictions, properties, and comparisons

Secondary structure predictions follow previous analyses (Seligmann and Raoult, 2016). Four variables are estimated from the optimal secondary structure predicted by Mfold (Zuker, 2003): 1. overall nucleotide percentage not involved in self-hybridization (loops); 2. percentage of nucleotides in closed loops at stem extremity among all nucleotides in loops (external loops); and 3. GC contents in loops, and 4. in stems. RNA size is not included among these variables.

**Figure 2:**
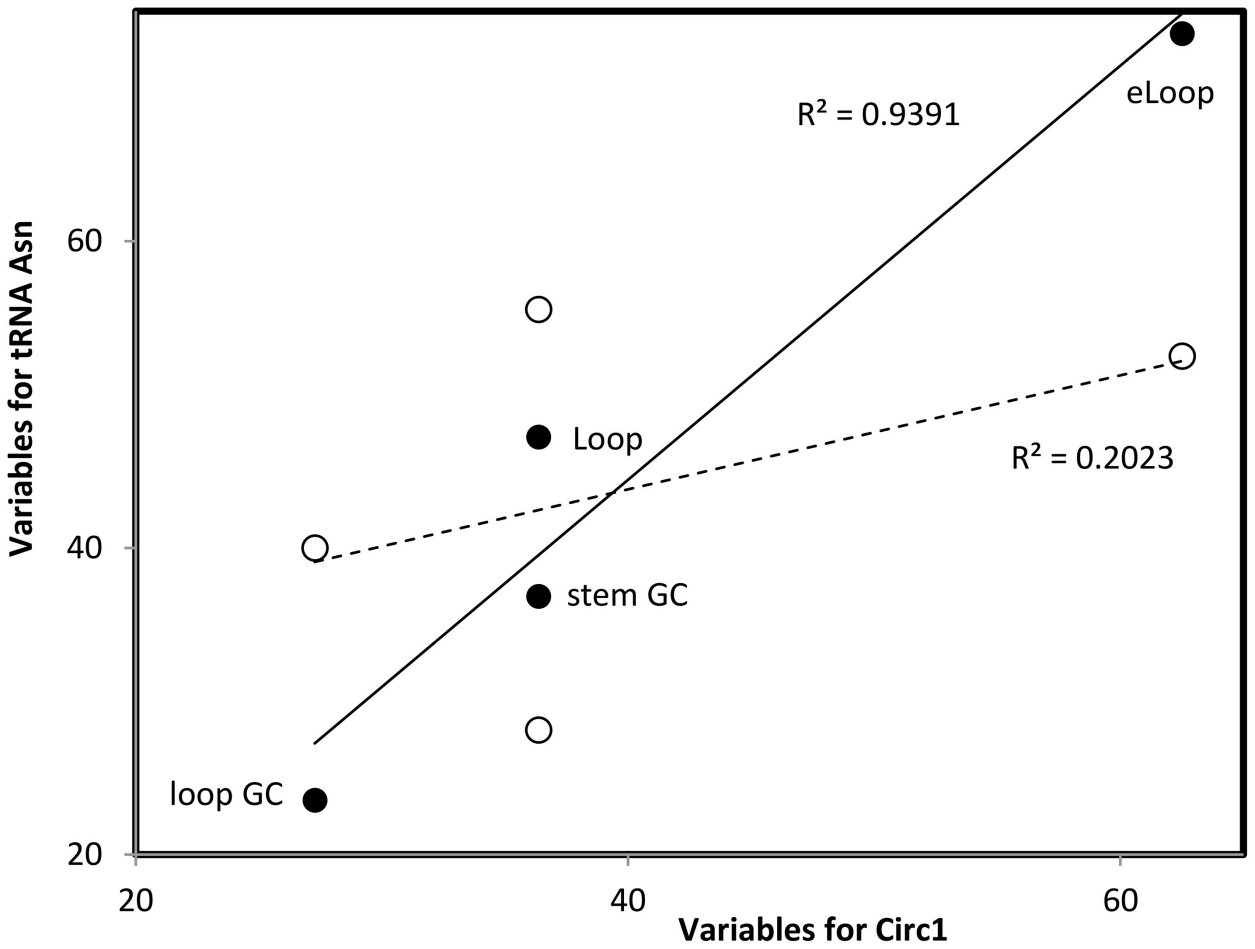
Multivariate comparisons between estimates of secondary structure variables for theoretical sequence Circ1 (from Table 1, x-axis) with corresponding variables for the cloverleaf (filled symbols, continous line) and OL-like (empty symbols, interrupted line) structures formed by the human mitochondrial tRNA Asn. Pearson correlation coefficients *r* = 0.969, *P* = 0.0; and *r* = 0.450, *P* < 0.05, respectively (values in the figure indicate the square of r). Such comparisons are calculated for each pair of secondary structures that are compared, RNAs are classified into secondary structure clusters (Figure 1) according to highest r.

Table 1 presents these variables for sequences analyzed here. They are compared with corresponding data from a previous collection of RNA sequences (Seligmann and Raoult, 2016, therein Table 1 and Figure 2) that defined clusters in Figure 1. Comparisons use Pearson's correlation coefficient r as a similarity estimate, setting statistically significant similarity at *r* = 0.95 (one tailed *P* = 0.05). Correlation analyses plot each of the four variables 1–4 of Y for one of the new RNAs analyzed here as a function of corresponding variables 1–4 of X for each of previously analyzed RNAs (Seligmann and Raoult, 2016, therein Table 1). Correlation coefficients estimate similarities between X and Y according to variables 1–4 (example in Figure 2). Results of statistical analyses presented here are validated also by the Benjamini-Hochberg correction method for false discovery rates which accounts for multiple tests (Benjamini and Hochberg, 1995; methodology detailed for unrelated analyses in Seligmann and Warthi, 2017). This test, unlike the classical Bonferroni approach that minimizes false positive detection rates and misses numerous positive results (Perneger, 1998) optimizes between false negative and false positive detection rates (Käll et al., 2007). The method is not affected by lack of independence between observations, and accounts for multiple testing. These additional analyses confirm classical statistics and therefore are not detailed in Results.

**Table 1:** Secondary structure properties of sequences analyzed here.

	***N***	**Loop**	**eLoop**	**Stem GC**	**Loop GC**	**Cluster**
Circ1	22	36.36	62.50	36.36	27.27	B1
Circ2	22	27.27	83.33	27.27	27.27	B1
Circ3	22	59.09	38.46	59.09	27.27	D2
Circ4	22	54.55	41.67	54.55	27.27	D2
Circ5	22	63.64	35.71	63.64	27.27	D2
Circ6	22	54.55	35.71	63.64	27.27	D2
Circ7	22	72.73	25.00	54.55	13.64	D2
Circ8	22	54.55	31.25	72.73	27.27	D1
Circ9	22	45.46	41.67	54.55	27.27	D2
Circ10	22	18.18	30.00	45.46	13.64	D2
Circ11	22	18.18	75.00	18.18	13.64	B1
Circ12	22	63.64	75.00	18.18	13.64	B1
Circ13	22	54.55	35.71	63.64	27.27	D2
Circ14	22	27.27	25.00	54.55	13.64	D2
Circ15	22	18.18	50.00	27.27	13.64	B1
Circ16	22	18.18	75.00	18.18	13.64	B1
Circ17	22	54.55	75.00	18.18	13.64	B1
Circ18	22	63.64	25.00	54.55	13.64	D2
Circ19	22	18.18	21.43	63.64	13.64	D1
Circ20	22	27.27	75.00	18.18	13.64	B1
Circ21	22	27.27	50.00	27.27	13.64	B1
Circ22	22	27.27	50.00	27.27	13.64	B1
Circ23	22	27.27	83.33	27.27	27.27	B1
Circ24	22	27.27	83.33	27.27	27.27	B1
*De novo*	51	16.67	40.00	56.00	60.00	B2
MITE16^*^	48	70.83	23.53	78.57	38.24	D1
MITE20^*^	66	48.48	28.13	58.82	28.13	D1
MITE4^*^	27	85.19	17.39	50.00	43.48	D2
MITE21^*^	60	85.00	54.90	90.00	35.29	D2
MITE14^*^	60	66.67	32.50	70.00	32.50	D2
MITE8^*^	57	68.42	53.85	100.00	30.77	D2
MITE2^*^	43	67.44	17.24	64.29	34.48	D2
MITE25^*^	62	54.84	38.24	64.29	20.59	D2
MITE9^*^	66	54.55	36.11	80.77	27.78	D1
MITE7^*^	57	68.42	30.77	50.00	33.33	D2
MITE12	68	64.71	22.73	87.50	31.82	D1
MITE29	62	70.97	34.09	94.44	27.27	D1
MITE28	71	61.97	20.45	84.62	31.82	D1
MITE24	64	59.38	23.68	50.00	28.95	D2
MITE19	67	71.64	22.92	80.00	35.42	D1
MITE17	65	73.85	29.17	87.50	37.50	D1
MITE10	72	52.78	47.37	82.35	36.84	D1
MITE26	62	67.74	26.19	70.00	26.19	D2
MITE13	63	68.25	76.74	95.00	27.91	D2
MITE22	62	64.52	32.50	75.00	35.00	D1
MITE5	45	60.00	37.04	68.75	33.33	D2
MITE11	41	56.10	26.09	88.89	21.74	D1
MITE23	62	70.97	34.09	83.33	34.09	D1
MITE1	61	60.66	37.84	58.33	37.84	D2
MITE15	61	67.21	31.71	75.00	29.27	D2
MITE6	36	61.11	36.36	57.14	40.91	D2
MITE3	57	63.16	22.22	54.55	33.33	D2
MITE18	62	61.29	42.11	75.00	26.32	D2
MITE27	62	64.52	25.00	60.00	32.50	D2
Mito 5S	44	36.36	37.50	14.29	0.00	B2
Mito 16S	46	52.17	25.00	4.46	29.17	D2
Mito 18S	59	66.10	28.21	45.00	20.51	D2
Mito 23S1	375	46.93	16.48	14.82	20.46	D2
Mito 23S2	276	43.12	19.33	29.30	23.53	D2
Mito 23S3	209	37.32	24.36	23.19	19.23	B2
Mimi 5S	41	75.61	25.81	10.00	16.13	B2
Mimi 16S	39	45.76	33.33	16.17	14.82	B2
Mimi 18S	59	52.54	12.90	17.86	12.90	D2
Mimi 23S1	380	48.95	19.36	11.60	19.36	D2
Mimi 23S2	349	39.26	23.36	21.91	25.55	B2
Mimi 23S3	207	34.78	22.22	18.06	12.50	B2
WNV 3′-5	105	20.00	19.05	54.02	33.33	D1
DENV 3′-5	105	27.62	24.14	50.00	27.59	D1
JEV 3′-5	111	23.13	13.79	51.22	27.59	D1
YFV 3′-5	107	23.37	20.00	57.32	36.00	D1
5′-UTR Dengue	123	49.59	34.43	26.02	45.90	D2
Surrounding	92	52.17	43.75	30.68	31.25	B2

Variables are: N- sequence length (not used for classifying RNAs in further analyses); Loop-percentage of nucleotides not involved in self-hybridization; eLoop-percentage of nucleotides among those in loops, in closed loops at stem extremity; percentage of GC in stems, and loops. “Cluster” indicates the cluster in Figure 1 with the highest similarity in secondary structure properties. “Circ” indicates sequences generated by simulations attempting to mimic circular RNA genesis (Demongeot and Moreira, 2007); “De novo” is a template-free synthesized sequence (Béguin et al., 2015); MITE1-29 are Pandoravirus'miniature inverted-repeat transposable elements from Submariner family (Sun et al., 2015),

* *indicates MITE inserted in a protein coding gene; Mito and Mimi are amoeban rRNA sequences aligning with Mimivirus sequences (alignments described in Table 3);” 5′-UTR” leader sequence of Dengue and West Nile virus (Gale et al., 2000); “Swinger alone” is the part of the 5′-UTR leader detected in Mimivirus' transcriptome, not templated by its genome; “surrounding” integrates the former swinger sequence with its untransformed surrounding Mimivirus sequences*.

### Mimivirus' RNA

*Mimivirus*' transcriptome (public data available at http://sra.dnanexus.com/studies/SRP001690/experiments; Legendre et al., 2010) are analyzed using CLCgenomicswb7. Reads are mapped on Mimivirus' reference genome NC_014649, according to the following criteria: at least half of the read maps to the reference genome, with at least 80% identity.

## Results

### Simulation-generated palindromes

Previous analyses produced the classification scheme of secondary structures formed by RNAs in Figure 1. Secondary structures formed by selected RNAs are analyzed to test this scheme. We classified the secondary structures of 24 sequences produced *in silico* by Demongeot and Moreira (2007). These RNAs were generated by simulations designed to reconstruct likely short primordial genes. Simulations were constrained to produce circular RNAs with codons coding for all 20 amino acids and a stop codon, and to form a stem-loop hairpin. The resulting theoretical RNAs have consensual tRNA sequence properties (Demongeot and Moreira, 2007).

Optimal secondary structures of these 24 theoretical RNAs (Circ1-24, Table 1) are compared with optimal secondary structures formed by RNAs used previously (Seligmann and Raoult, 2016, therein Table 1) as shown for Circ1, in Figure 2. The secondary structure of Circ1 resembles the cloverleaf structure of tRNA Asn, and much less the OL-like structure formed by that tRNA sequence. Mitochondrial tRNAs form secondary structures that resemble mitochondrial light strand replication origins (OL), presumably function as OLs in OL absence (Desjardins and Morais, 1990; Seligmann and Labra, 2014).

Half of the 24 theoretical RNAs designed by simulations cluster with B1. Ten cluster with D2 (as shown in Figure 2), and the remaining two with D1. There are 24 × 6 = 144 comparisons between the 24 simulation-generated RNAs and the six RNAs in B1. Of these comparisons, 19 (13.2%) have Pearson correlation coefficients r with *P* < 0.05. Among the 24 × 17 = 408 comparisons with D2, 10 (2.45%) have *P* < 0.05. There are 24 × 13 = 312 comparisons with D1; only two (0.64%) have *P* < 0.05. No comparison between the 24 simulation-generated RNAs with D2 has *P* < 0.05. Hence our interpretation of B1 as representing secondary structures formed by ancestral RNAs agrees with the independent approach developed by Demongeot and Moreira (2007).

The fact that secondary structures formed by the 24 simulation-generated, presumably ancestral-like RNAs of Demongeot and Moreira (2007) preferentially cluster with B1 confirms the ancestral status of these theoretical RNAs and of cluster B1. The observation that translation/genetic code properties converge with tRNA sequence properties (Demongeot and Moreira, 2007) is also congruent with analyses of tRNAs along the principles of the natural circular code (Michel, 2012, 2013; El Soufi and Michel, 2014, 2015): a set of 20 codons overrepresented in coding vs. other frames of protein coding genes (Arquès and Michel, 1996), which enable coding frame retrieval (Ahmed et al., 2007, 2010; Michel and Seligmann, 2014). Results also fit a model of evolution of secondary structures into sequence signals such as codons (El Houmami and Seligmann, 2017).

### Template-free synthesized sequence

Simulation-generated sequences such as those in the previous section are suboptimal to confirm the ancestral/*de novo* status of clusters B1/B2. We analyze the predicted optimal secondary structure formed by a short sequence that is synthesized template-free by a combination of three archaeal enzymes, including DNA polymerase PolB (Béguin et al., 2015). This sequence is by definition “*de novo*.” Its secondary structure properties most resemble those of an exceptional, protein-encoding viroid in cluster B2 (one tailed *P* = 0.034). This result is statistically significant also after considering multiple testing, using the Benjamini-Hochberg correction for false discovery rates that accounts for the number of tests done (Benjamini and Hochberg, 1995). This strengthens the status of cluster B2 as recent spontaneously generated RNAs.

### Ancient and *de novo* viroids

Evidence for recent vs. precellular origins of viroids are equivocal. Potentially, results of previous classifications of viroid secondary structures might be biased by including in analyses viroids with high GC contents and forming complex branching secondary structures. Analyses here include ten viroids forming rod-like secondary structures with GC contents ranging from 35 to 61%. All cluster with D1 and D2 (seven and three viroids, respectively, Table 2). No comparison with B1 and B2 has *P* < 0.05. Among 130 and 170 comparisons of these 10 rod-shaped viroids with secondary structures belonging to D1 and D2, 47 and 8 (36.2 and 4.7%, respectively), have *P* < 0.05.

**Table 2:** Secondary structure variables and classification of 10 viroids forming rod-shaped secondary structures.

**Viroid**	***N***	**Loop**	**eLoop**	**Stem GC**	**Loop GC**	**Cluster**
CVd IV^a^	284	28.87	9.76	64.36	36.37	D1
TPMV^b^	360	33.06	10.08	36.37	42.02	D2
TASV^b^	360	28.33	11.77	60.02	34.31	D1
ASBV^c^	359	29.81	7.48	63.49	42.06	D1
PTSV^c^	247	32.39	17.50	40.12	31.25	D2
CSV^c^	366	28.96	6.60	55.39	41.51	D2
PBCVd^d^	315	31.43	31.32	71.76	37.37	D1
ADFVd^e^	310	34.52	11.22	61.58	37.37	D1
CEVd^f^	371	30.46	7.97	66.67	46.90	D1
HSVd^f^	302	31.46	8.42	66.67	33.68	D1

*Secondary structures are according to corresponding references 1–6. All rod-shaped viroids, independently of GC contents, group with 23S (D1) and 16S (D2) rRNAs, indicating ancient origins*.

a Citrus viroid IV (Puchta et al., 1991);

b Tomato planta macho viroid, tomato apical stunt viroid (Kiefer et al., 1983);

c Avocado sunblotch viroid, Potato spindle tuper viroid, Chrysanthemum stunt viroid (Symons, 1981);

d Pear blister canker viroid (Hernandez et al., 1992);

e Apple dimple fruit viroid, (Chiumenti et al., 2014);

f *Citrus exocortis viroid, Hop stunt viroid (Lin et al., 2015)*.

Hence rod-shaped viroids belong to the ancestral tRNA-rRNA axis of molecular evolution. Viroids with more complex branching patters clustered with B2 according to previous analyses (Seligmann and Raoult, 2016). This suggests that viroid ‘survival’ requires evolutionary secondary structure simplification, perhaps because endonucleases target secondary branching (Fujishima et al., 2011). Results are compatible with mixed evidence for ancient precellular origins and recent *de novo* emergence of viroids.

### Pandoravirus' miniature inverted-repeat transposable elements, mite

Genomes of giant viruses include many inverted repeats forming stem-loop hairpins regulating transcription (Byrne et al., 2009; Claverie and Abergel, 2009), reminiscent of mitochondrial posttranscriptional tRNA punctuation (Ojala et al., 1981). These include miniature inverted-repeat transposable elements, MITEs (Fattash et al., 2013). The MITE family submarine in the giant virus *Pandoravirus salinus* presumably invaded that genome relatively recently (Sun et al., 2015).

We classified optimal secondary structures formed by these 29 MITE submarine sequences with clusters in Figure 1. No submarine MITE clusters within B1 or B2, but 12 cluster within D1 [11 among 377 comparisons (2.9%) with *P* < 0.05] and 17 cluster within D2 [30 among 493 comparisons (6.1%) with *P* < 0.05]. The 10 submarine MITE sequences integrated in Pandoravirus' protein coding genes cluster slightly more frequently with D2 than D1, as compared to the remaining submarine MITEs (difference not statistically significant). Hence most Pandoravirus submarine MITEs cluster with D2 (characterized by bacterial RPEs and 16S rRNA subunits), resembling RNAs from the main tRNA-rRNA axis. Under this scenario, similarities with D1 (characterized by retroviruses and 23S rRNA subunits) would result from chance or secondary convergences. However, this interpretation does not account for additional lateral transfers: many genes of giant viruses originate from lateral transfers, mainly from bacteria sharing their habitat in their amoeban host (Moliner et al., 2010; Georgiades and Raoult, 2012).

### Ribosomal RNA-like mimivirus sequences

We used blastn (Altschul et al., 1997) to explore putative links between ribosomal RNAs and giant virus genomes. For that purpose we extracted rRNA sequences from *Acanthamoeba castellanii*'s complete mitochondrial genome (NC_001637) and aligned these with *Acanthamoeba polyphaga* Mimivirus' complete genome (NC_014649). The amoeba's mitogenome includes four rRNA genes—5S, 16S, and 18S rRNAs—and a 23S-like sequence (Burger et al., 1995). Blastn alignment criteria were set at the shortest word size (7), the weakest match/mismatch scores (1/−1) and gap costs (existence 0; extension 2). For each rRNA we chose the alignment with the lowest (best) e value among the alignments detected by blastn between these mitochondrial rRNA genes and Mimivirus' genome (Table 3).

**Table 3:** Sequences aligned between *Acanthamoeba castellani*'s mitochondrial rRNA genes and *Acanthamoeba polyphaga* Mimivirus' genome.

**rRNA**	**E value**	**Mito 5′-3′**	**Mimivirus 5′-3′**
5S	0.001	58–101	768283–768243
18S	0.069	104–162	23259–23317
16S	0.009	1103–1148	759756–759718
23S1	0.0007	3–377	500064–500442
23S2	0.002	962–1291	175514–175166
23S3	0.0003	1970–2181	89598–89391

For 23S-like rRNA, three alignments are considered because they represent similarities with different Mimivirus sequences and the alignments have low e values. Secondary structures formed by four of the six rRNA sequences aligning with Mimivirus sequences cluster best with D2 and two sequences cluster best with B2 (Figure 1). Hence these rRNA sequences most resemble the cluster that includes bacterial 16S rRNA subunits.

These rRNA sequences aligned with sequences from the Mimivirus genome. These Mimivirus sequences also form secondary structures which cluster differently in Figure 1 than their putative amoeban mitochondrial rRNA homologs. Optimal secondary structures formed by four of the six corresponding Mimivirus DNA sequences cluster best with B2 and the remaining two with D2. Very few of the r coefficients used for these classifications have *P* < 0.05. Hence these results must be considered as tentative.

Differences in clustering by secondary structures formed by mitochondrial rRNA sequences vs. Mimivirus' genome for aligning sequence pairs suggest that alignments are frequently due to convergences between rRNAs and viral sequences. A possible interpretation of these clustering results (Table 1) is that viruses tend to create *de novo* rRNA-like sequences, though some of the alignments might suggest regular homology due to common ancestry. Lateral transfers between the host and Mimivirus is also a reasonable explanation. Independently of lateral transfers, this putative mitochondrial rRNA-Mimivirus homology is in line with common ancestry between Megavirales and a cellular ancestor of mitochondria, as suggested by homologies between polymerases of these organisms (Kempken et al., 1992; Rohe et al., 1992; Kapitonov and Jurka, 2006; Yutin et al., 2013; Krupovic and Koonin, 2016; Koonin and Krupovic, 2017) and above noted similar regulations of posttranscriptional processing (vertebrate mitochondria, Ojala et al., 1981; Claverie and Abergel, 2009; Mimivirus, Byrne et al., 2009). Hence secondary structure analyses apparently strengthen the hypothesis that mitochondria share a common ancestor with Megavirales.

### Flavivirid virus leading regions

Analysis of conserved secondary structures formed by 3′ and 5′ RNA structures in four flavivirid viruses [Dengue (DENV), West Nile (WNV), Japanese encephalitis (JEV) and tick-borne encephalitis (YFV) viruses, Brinton and Basu, 2015; therein Figure 1B, secondary structure variables here in Table 1] show that these structures cluster with D1 (characterized by 23S rRNA subunits). Hence these Flavivirus sequences crucial to replication form structures that resemble 23S rRNA, as observed for most rod-shaped viroids (section Ancient and *de novo* Viroids). Most Pandoravirus MITE sequences resemble D2, characterized by 16S rRNA (section Pandoravirus' Miniature Inverted-Repeat Transposable Elements, Mite) and some Mimivirus sequences resembling mitochondrial rRNAs cluster within D2 (section Ribosomal RNA-Like Mimivirus Sequences). Overall results indicate the hypothesized link between viral RNAs and rRNAs.

### A template-free RNA in mimivirus' transcriptome persists during infection

Mimivirus' transcriptome [data from (Legendre et al., 2010), available at: http://sra.dnanexus.com/studies/SRP001690/experiments] includes numerous short RNAs that are not homologous to Mimivirus' genome. Here we focus on a 42-nucleotide-long sequence (5′-GAGACACGCAACAGGGGATAGGCAAGGCACACAGGGGATAGG-3′) because this sequence also occurs according to Blastn in diverse taxa: Megavirus, Dengue and West Nile genomes, copperhead snake microsatellites and levant cotton repeats. This Mimivirus RNA matches the giant virus Megavirus terra1 (KF527229, positions 903759-903800) and some (not all) genomes of Dengue and West Nile viruses (both are Flaviviridae). In these Flaviviridae, the sequence is inserted in (or close to) the 5′ UTR leading region. The e value of alignments between Mimivirus' RNA and the flavivirid 5′ UTR sequences is 2 × 10^−11^. This RNA is detected by Blastn (word size 7; Match/Mismatch scores 1,−1; Gap costs, existence 1, extension 1) in Mimivirus' transcriptome throughout amoeban infection: −15, 0, 90, 180, 360a,b, 540, and 720 min after infection. This RNA does not map on any region of Mimivirus' genome.

### Potential origins of mimivirus' template-free RNA

This RNA not templated by Mimivirus' genome might originate from accidental contamination. However, three arguments suggest less conventional hypotheses. Firstly, this RNA is repeatedly detected throughout the virus' infection cycle, hence in different sequencing events. Secondly, the exact same RNA is detected each time in terms of sequence and length. Thirdly, this exact sequence occurs in the genome of another giant virus, Megavirus terra1. These arguments suggest that the occurrence of this RNA in Mimivirus' transcriptome is not circumstantial.

This RNA could originate from (a) *de novo* creation, as suggested for some short stem-loop hairpin RNAs (Seligmann and Raoult, 2016) and analyses in previous sections, (b) pools of vertically transmitted RNAs originating from horizontal transfers (Stedman, 2013) forming quasi species groups (Villarreal, 2015, 2016), or (c) noncanonical transcriptions of genomic DNA, such as RDDs [RNA-DNA differences, which result from posttranscriptional nucleotide substitutions (Li et al., 2011) or indels (insertions/deletions, Chen and Bundschuh, 2012)]. In addition, this RNA might result from a peculiar type of transcription, which produces transcripts matching genomes only if one assumes that transcription systematically exchanges nucleotides over the whole length of the transcript, which is called swinger transcription. There are 23 possible nucleotide exchange rules, 9 are symmetric exchanges of type X<->Y and 14 are asymmetric exchanges of the type X->Y->Z->X. These 23 transformations are each separately applied *in silico* to Mimivirus' genome to produce 23 swinger-transformed versions of that genome which are used for further analyses. Current information on systematic nucleotide exchanges is reviewed by Seligmann (2017), with further references therein (see also Seligmann, 2013; Michel and Seligmann, 2014).

### Swinger transcript or template free RNA polymerization?

**Figure 3:**
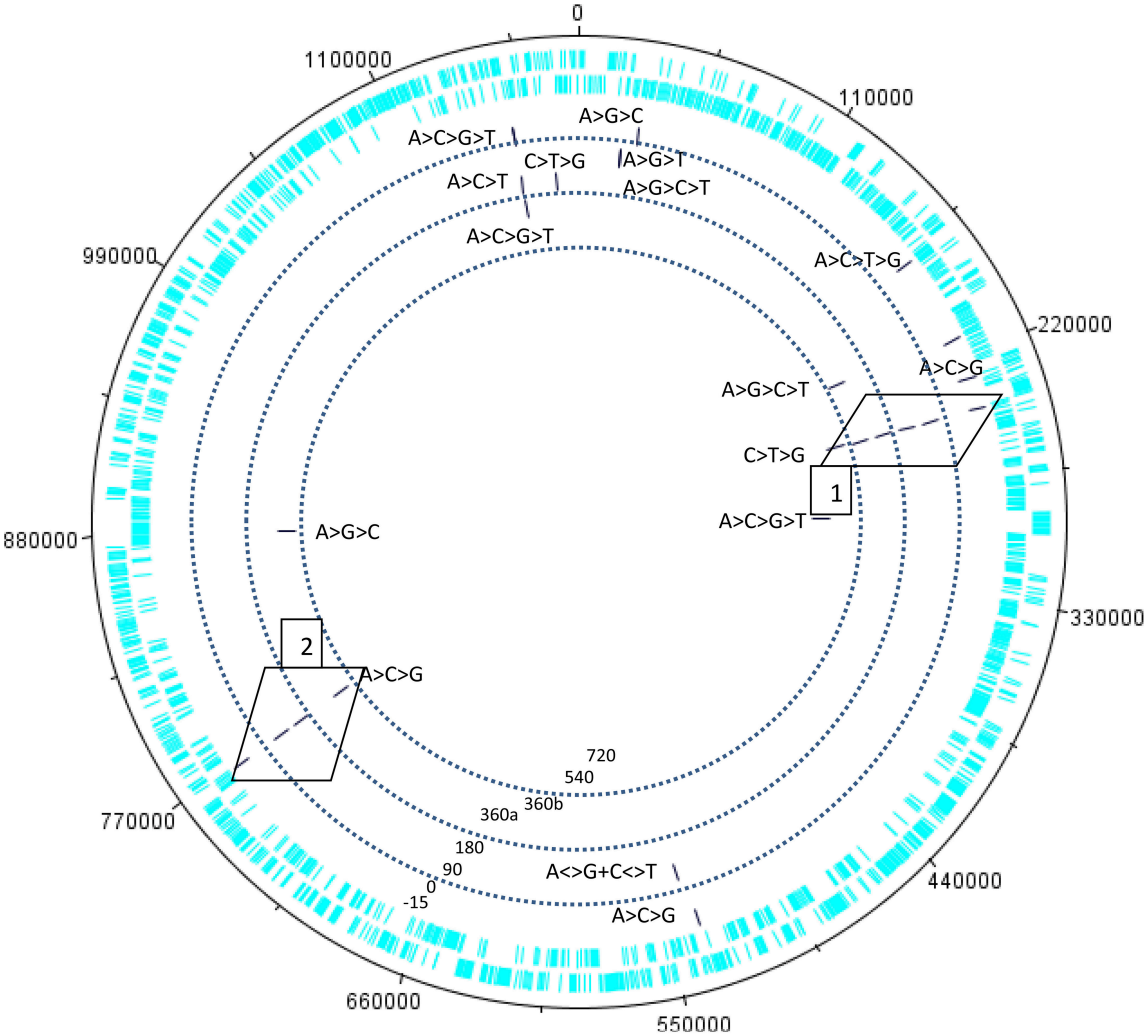
Kinetics of the swinger transcriptome (asymmetric nucleotide exchanges) of *Mimivirus*. Concentric circles indicate different periods in the cycle of *Mimivirus*. Bars indicate swinger RNAs detected for a given genomic region, the swinger transformation rule is indicated next to the bar. Swinger RNAs in boxes 1 and 2 are the exact inverse complements of each other, and correspond to the 5′ UTR leading region of Dengue and West Nile viruses.

**Figure 4:**
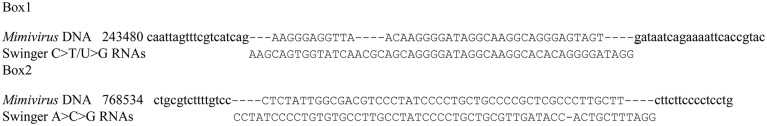
Alignment between sequence in **Boxes 1** and **2** (Figure 3) and homologous sequences: *Mimivirus* reference genome sequence.Underlined are the detected swinger RNA sequences. Small caps indicate neighboring sequences that are not transformed by nucleotide exchange.

**Figure 5:**
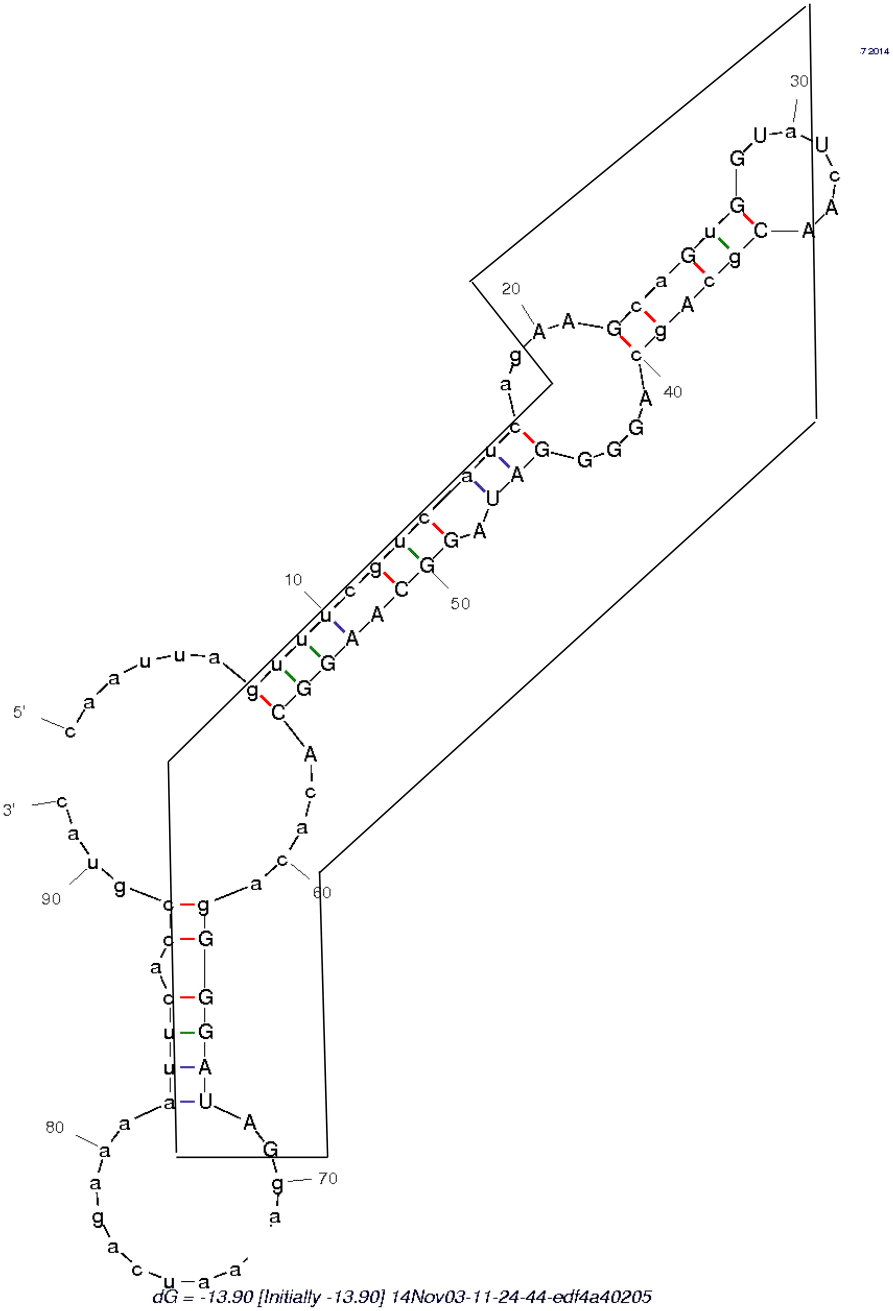
Secondary structure formed by the swinger C>T/U>G RNA (in box) and its untransformed neighboring regions.

Our preliminary explorations of the kinetic data of the transcriptome of the giant virus Mimivirus (Raoult et al., 2004; Legendre et al., 2010, 2011) detected putative swinger RNAs among RNAs that do not match the regular genome sequence, after excluding regular RNAs by mapping on the regular genome (Figures 3–**5**). Table 4 compares abundances and mean lengths of detected putative swinger RNAs among transcriptomic data produced by 454 and SOLID massive sequencing techniques (SOLID data unpublished, available in our laboratory). Results by both techniques are comparable. Abundances estimated from 454 sequencing correlate positively with those produced by SOLID (Spearman rank nonparametric correlation rs = 0.323, one tailed *P* = 0.066). Similarly, mean lengths of swinger reads correlate positively for data produced by 454 and SOLID (rs = 0.394, one tailed *P* = 0.082). Combining *P*-values from these two tests using Fisher's method for combining P values (Fisher, 1950), which sums −2 × ln(Pi) where i ranges from 1 to k tests (here *k* = 2) yields a chisquare statistic with 2 × k degrees of freedoms with a combined *P* = 0.034. Hence results from both sequencing methods are overall congruent, swinger RNAs are not artifacts resulting from massive sequencing technologies.

**Table 4:** Swinger transformations of the genome of Mimivirus, and swinger transcripts.

	**454**		**Solid**	
**Swinger**	***N***	**Mean**	***N***	**Mean**
A<->C	5	51.40	7261	35.52
A<->G	8	211.63	5294	36.47
A<->T	0		7234	35.99
C<->G	173	196.74	7314	35.92
C<->T	12	199.58	5800	36.29
G<->T	1	56.00	5553	35.63
A<->C-G<->T	0		2634	36.60
A<->G-C<->T	1	68.00	2672	36.37
A<->T-C<->G	0		1805	37.14
A->C->G->A	9	62.11	6238	35.73
A->C->T->A	1	64.00	4963	36.64
A->G->C->A	2	70.50	5013	36.68
A->G->T->A	1	79.00	6127	35.70
A->T->C->A	0		6325	35.97
A->T->G->A	0		4826	36.72
C->G->T->C	0		4856	36.68
C->T->G->C	9	52.67	5962	35.70
A->C->G->T->A	4	84.50	6007	36.28
A->C->T->G->A	1	77.00	8421	35.83
A->G->C->T->A	2	74.50	7675	35.45
A->G->T->C->A	0		8444	35.85
A->T->C->G->A	0		5289	35.71
A->T->G->C->A	0		5429	36.41

*Columns are: 1–2. Number and mean length (nucleotides) of swinger RNAs matching the Mimivirus' genome swinger version over>half of its length with >80% identity, 454 sequencing, all times since infection pooled (Legendre et al., 2011); 3–4. Number and mean length (nucleotides) of swinger RNAs matching the Mimivirus' genome swinger version over >half of its length with>80% identity, SOLID sequencing, at 16 h post infection*.

Within 454 data, two swinger RNAs (boxes in Figure 4, primary and secondary structures in Figures 4, 5) were detected throughout most of the viral cycle, corresponding to genomic sequences at positions 243499-243537 (C->T->G->C swinger rule, box 1 in Figure 3) and 768549-768596 (A->C->G->A swinger rule, box 2 in Figure 4). These two genomic positions are each other's inverse complement. This is also the case for the corresponding swinger RNA reads. These reads correspond to the aforementioned template-free RNA and are homologous with Megavirus terra1 (KF527229: positions 903764-903800). This candidate swinger RNA aligns only with swinger transformed versions of Mimivirus' genomic sequence, but its similarity with the swinger transformed genome is also low.

Putatively, some genomic sequences in *Mimivirus* could originate from horizontal transfers from other viruses, suggesting a potential implication in the horizontal transfer of the Sputnik virophage that infests *Mimivirus* (La Scola et al., 2008). This is in line with the chimeric origin of most *Mimivirus* genes (originating from eukaryotic hosts and bacterial co-parasites of the eukaryotic host, Moreira and Brochier-Armanet, 2008; Jeudy et al., 2012), and confirms horizontal transfer of other viral sequences (Yutin et al., 2013), via swinger transformations.

## General comments

Analyses that integrate molecular structure information are surprisingly successful at resolving various biological problems, notably of ancient evolution (for example the presumed monophyletic cellular origin of viruses, Nasir and Caetano-Anollés, 2015). However, techniques enabling this remain relatively inaccessible, notably for RNAs. This situation is particularly true if analyzes are supposed to integrate nonequilibrium dynamics of folding during the synthesis of the molecule [proteins: cotranslational folding, (Holtkamp et al., 2015; Seligmann and Warthi, 2017); RNAs: cotranscriptional folding, Gong et al., 2017]. Indeed, most RNAs fold during their syntheses, the new fold appearing after a new stretch of nucleotides is added to the elongating RNA (Schroeder et al., 2002). Each new fold is partially constrained by the previous one, which renders prediction algorithms more complex (Zhao et al., 2010; Frieda and Block, 2012).

The approach used here does not require any special computational skills. It could be adapted to dynamical contexts, and to include information on groups of closely related, suboptimal secondary structures, when these are only slightly more unstable than the optimal structure. Previous analyses (Seligmann and Raoult, 2016) included such special cases, for mitochondrial tRNAs in their classical cloverleaf fold, and in their replication origin (OL)-like fold. Analyzes separating different folds with similar stabilities for structures formed by the same sequence, such as cloverleaf vs. OL-like tRNA structures, yield sometimes different results in classifications such as that in Figure 1 (see Seligmann and Raoult, 2016, therein Figure 2).

In addition, molecules with very similar estimates for all variables may form very different structures, or similar structures of very different sizes (the variables do not include sequence length). Hence the simple approach used here could be applied to study closely related clouds of secondary structures formed by a given RNA. It could also be adapted to discriminate further between similar secondary structures by including additional variables, such as GC contents in internal vs. external loops, and angular rotation between stem branches.

Overall our results indicate that the versatility of RNA structures enable for functional novelties. Their physicochemical properties are also compatible with this role in primitive protolife organic conditions.

## Conclusions

Interpretations of a previous classification of RNA secondary structures formed by a variety of RNAs (Seligmann and Raoult, 2016) were tested by classifying specific RNAs of special interest. For example, circular RNAs generated by simulations presumably mimicking ancestral RNAs cluster mainly, as expected, with cluster B1, a presumed group of ancestral RNAs (Figure 1).

Cluster B2 was previously interpreted as representing *de novo* emerged RNAs because it grouped short simple RNAs from very different functional types (tRNAs, ribozymes, viroids, etc.). Secondary structures formed by a sequence synthesized template free (hence *de novo*) cluster with B2.

Rodshaped viroids from a wide range of GC contents belong independently of GC contents to D1 and D2, two clusters characterized by rRNAs and parasitic sequences. Viroids forming secondary structures characterized by more complex branching patterns belong to cluster B2 (putative *de novo* RNAs). Hence results suggest that some viroids are recent, rodshaped ones are presumably ancient RNAs.

Presumed parasitic palindromic sequences from Pandoravirus (MITE submarine family) resemble cluster D2. D2 is characterized by16S rRNA subunits and Rickettsial palindromic elements that parasitize *Rickettsia* genomes. This fits previous grouping of secondary structures formed by parasitic sequences with 23S and 16S rRNAs (clusters D1 and D2).

Mimivirus sequences that align with amoeaban mitochondrial rRNA genes also strengthen suspected evolutionary links between rRNA and viral sequences. Mitochondrial rRNA sequences aligning with Mimivirus sequences cluster as expected with D2, characterized by bacterial 16S rRNA. Interestingly, secondary structures formed by Mimivirus sequences with which the mitochondrial rRNAs align, cluster mainly with B2, suggesting recent *de novo* origins for viral sequences resembling rRNAs. These results are based on relatively weak similarities and hence can only be considered as preliminary. Nevertheless, they suggest that viruses produce rRNA-like sequences, in line with the prediction that the study of giant viruses will ‘change current conceptions of life, diversity and evolution’ (Abrahao et al., 2014).

The secondary structure formed by the 5′ UTR leading region of flavivirid viruses clusters also with D2, hence it is a further viral, rRNA-like sequence. An RNA persisting throughout Mimivirus' infection cycle and lacking homology with Mimivirus' genome occurs also in some flavivirus genomes, Megavirus, in copperhead snakes and levant cotton. This saltatory phylogenetic distribution is compatible with repeated spontaneous, template free synthesis by polymerases deterministically producing specific sequences, as observed in Archaea (Béguin et al., 2015). Indeed, this RNA, embedded in the surrounding regular 5′ and 3′ sequences (Table 1 and Figure 5), forms a secondary structure that clusters with B2. This would suggest *de novo* emergence of this unusual RNA.

Analyses assuming different scenarios based on noncanonical transcriptions do not reach clear-cut conclusions on the origin of that RNA that does not map to the Mimivirus genome. Though contamination cannot be totally excluded, other hypotheses seem plausible. Indeed, this RNA maps imperfectly on Mimivirus' swinger-transformed genome (Mimivirus' transcriptome includes numerous swinger RNAs, results from each 454 and SOLID massive sequencing methods are congruent). Overall, results hint that parasitic RNAs form rRNA-like secondary structures, and template free polymerizations apparently enrich genomes with new RNA/DNA sequences.

## Author contributions

HS and DR designed the study and analyses.

### Conflict of interest statement

The authors declare that the research was conducted in the absence of any commercial or financial relationships that could be construed as a potential conflict of interest.
